# Estrogen increases ENaC activity via PKC*δ* signaling in renal cortical collecting duct cells

**DOI:** 10.14814/phy2.12020

**Published:** 2014-05-28

**Authors:** Yamil R. Yusef, Warren Thomas, Brian J. Harvey

**Affiliations:** 1 Department of Molecular Medicine, Royal College of Surgeons in Ireland, RCSI Education and Research Centre, Beaumont Hospital, Dublin, Ireland

**Keywords:** ENaC, kidney, estrogen, PKC*δ*

## Abstract

The most active estrogen, 17*β*‐estradiol (E2), has previously been shown to stimulate a female sex‐specific antisecretory response in the intestine. This effect is thought to contribute to the increase in whole body extracellular fluid (ECF) volume which occurs in high estrogen states, such as in the implantation window during estrous cycle. The increased ECF volume may be short‐circuited by a renal compensation unless estrogen exerts a proabsorptive effect in the nephron. Thus, the effect of E2 on ENaC in kidney cortical collecting duct (CCD) cells is of interest to understand estrogen regulation of ECF volume. Previous studies showed a rapid stimulatory effect of estrogen on ENaC in bronchial epithelium. In this study we examined if such a rapid effect on Na^+^ absorption could occur in the kidney. Experiments were carried out on murine M1‐CCD cell cultures. E2 (25 nmol/L) treatment caused a rapid‐onset (<15 min) and sustained increase in the amiloride‐sensitive Na^+^ current (I_Na_) in CCD monolayers mounted in Ussing chambers (control, 1.9 ± 0.2 *μ*A/cm^2^; E2, 4.7 ± 0.3 *μ*A/cm^2^; *n* = 43, *P* < 0.001), without affecting the ouabain‐sensitive Na^+^/K^+^ pump current. The I_Na_ response to E2 was inhibited by PKC*δ* activity antagonism with rottlerin (5 *μ*mol/L), inhibition of matrix metalloproteinases activity with GM6001 (1 *μ*mol/L), inhibition of EGFR activity with AG1478 (10 *μ*mol/L), inhibition of PLC activity with U‐73122 (10 *μ*mol/L), and inhibition of estrogen receptors with the general ER antagonist ICI‐182780 (100 nmol/L). The estrogen activation of I_Na_ could be mimicked by the ER*α* agonist PPT (1 nmol/L). The nuclear excluded estrogen dendrimer conjugate (EDC) induced similar stimulatory effects on I_Na_ comparable to free E2. The end target for E2 stimulation of PKC*δ* was shown to be an increased abundance of the *γ*‐ENaC subunit in the apical plasma membrane of CCD cells. We have demonstrated a novel rapid “nongenomic” function of estrogen to stimulate ENaC via ER*α*‐EGFR transactivation in kidney CCD cells. We propose that the salt‐retaining effect of estrogen in the kidney together with its antisecretory action in the intestine are the molecular mechanisms causing the expanded ECF volume in high‐estrogen states.

## Abstract (precis)

Estrogen stimulates sodium absorption in kidney cells. This rapid “nongenomic” response to estrogen is transduced via estrogen receptor transactivation of the epidermal growth factor receptor. The ER‐EGFR transactivation triggers a protein kinase signaling cascade which culminates in the insertion of sodium channel subunits into the cell membrane. Estrogen is a novel salt‐retaining hormone with proabsorptive effects in kidney and antisecretory actions in intestine.

## Introduction

17*β*‐estradiol (E2) is the major estrogen naturally occurring in the female body. Our laboratory has provided evidence showing that E2 produces an antisecretory effect in the distal colon of female rats (O'Mahony et al. 2007). The antisecretory response to E2 has a short‐term (nongenomic) and a long‐term (genomic) component. The short‐term component involves activation of protein kinase C delta (PKC*δ*) and subsequent inhibition of the basolateral K^+^ channel KCNQ1:KCNE3 and the long‐term component involves changes in the trafficking of KCNQ1 and the expression of transporter proteins necessary for Cl^−^ secretion (O'Mahony et al. 2009). The antisecretory response to E2 could provide an explanation for salt and fluid retention observed in females during states of high circulating levels of estrogen during phases of the estrous cycle (Crocker 1971) and pregnancy. Extracellular fluid expansion has physiological relevance for the endometrial swelling and restructuring that occurs in advance of embryo implantation and could also impact upon cardiovascular function.

The antisecretory effects of E2 in the intestine may be short‐circuited by compensatory Na^+^ and water excretory mechanisms in the kidney and it has been proposed but not yet confirmed that estrogen may exert a proabsorptive effect in this organ.

In the kidney, specifically at the aldosterone‐sensitive distal nephron (ASDN), the reabsorption of Na^+^ is mediated by the activity of the epithelial Na^+^ channel (ENaC). This channel belongs to the ENaC/degenerin family of ion channels and is composed of three structurally related subunits termed *α*,* β*, and *γ* (Rossier and Stutts 2009). ENaC is expressed at the apical membrane of the principal cells in the ASDN and the channel activity is regulated by hormones such as vasopressin and aldosterone (Lee et al. 2008; Bubien 2010; Butterworth 2010; Thomas and Harvey 2011). There is compelling evidence suggesting that the activity of ENaC could also be regulated by E2 in the kidney. In ovariectomized rats, E2 raises plasma Na^+^ concentration (Zheng et al. 2006) and increases renal *α*‐ and *β*‐ENaC subunit mRNA levels (Gambling et al. 2004; Riazi et al. 2006). In mouse collecting duct cells, E2 increases the activity of ENaC (Chang et al. 2007). Strengthening this idea, similar effects have been described in nonrenal cell types such as rat alveolar epithelial cells and osteoblasts, where E2 increases the activity, mRNA and protein abundance of ENaC (Sweezey et al. 1998; Laube et al. 2011; Yang et al. 2011; Greenlee et al. 2013).

On the basis of this evidence, we reasoned that in addition to the gender‐specific antisecretory effect of E2 observed in distal colon, a putative proabsorptive effect of E2 in the kidney, specifically in the CCD cells, could provide an additional mechanism to explain salt and fluid retention observed in females during periods of high circulating levels of E2.

To test the hypothesis for a proabsorptive effect of E2 we used the renal cortical collecting duct M1 cell line (M1‐CCD) as a model for Na^+^‐absorbing principal cells. We investigated the effect of E2 on the amiloride‐sensitive short‐circuit current, ENaC subunit trafficking and potential protein kinase regulatory signaling pathways.

## Materials and Methods

### Cell culture

The M1 cortical collecting duct cell line (M1‐CCD) was obtained from ATCC (CRL‐2038). This cell line was derived from renal CCD tubules microdissected from a transgenic mouse containing the early region of SV40 virus (strain Tg [SV40E] Bri7) (Stoos et al. 1991). M1‐CCD cells were cultured in 75 cm^2^ or 25 cm^2^ polystyrene flasks containing a mixture of 1:1 Dulbecco's modified Eagle's medium and Ham F‐12 medium (DMEM:F‐12) with fetal bovine serum (5%); supplemented with l‐glutamine (2 mmol/L), penicillin (100 *μ*/mL)/streptomycin (100 *μ*g/mL), and dexamethasone (5 *μ*mol/L). Cells were maintained in atmosphere of 5% CO_2_, 37°C, and 70% humidity. Culture medium was changed every 3 days and cells were subcultured when they became 70% confluent. M1‐CCD cells were maintained in serum‐free medium and in the absence of dexamethasone overnight before treatment with E2.

### Western blotting

Cell lysates were dissolved by boiling in Laemmli sample buffer (Sigma‐Aldrich Ireland Limited, Arklow, Ireland) and proteins were separated by SDS‐PAGE on 8% (w/v) polyacrylamide gels. The proteins were transferred onto nitrocellulose membranes, and probed with the specified primary antibodies diluted in 5% (w/v) skimmed milk or bovine serum albumin (BSA) in TBS 0.1% Tween20, according to the supplier's instructions. Primary antibodies used were mouse anti‐*β*‐actin (dilution 1:5000) (Sigma‐Aldrich Ireland Limited), rabbit anti‐phospho‐PKC*δ* (Ser643) (dilution 1:1000) (Cell Signaling Technology, Brennan & Co., Dublin, Ireland), rabbit anti‐*α*‐ENaC (dilution 1:500) (Millipore, Millipore Cork, Ireland, Cat. # AB3530P), rabbit anti‐*β*‐ENaC (dilution 1:500) (Abcam, Cat. # ab2906), rabbit anti‐*γ*‐ENaC (dilution 1:500) (Abcam, Mayo, Ireland, Cat. # ab3468), mouse antiestrogen receptor *α* (dilution 1:1000) (Cell Signaling Technology), and rabbit antiestrogen receptor *β* (dilution 1:1000) (Cell Signaling Technology). Bound antibodies were detected using anti‐rabbit IgG, HRP‐linked (dilution 1:5000) (Cell Signaling Technology) or anti‐mouse IgG, HRP‐linked (dilution 1:5000) (Cell Signaling Technology); antibody‐labeled proteins were visualized by enhanced chemiluminescence, ECL+ (Amersham Biosciences, Buckinghampshire, UK). Exposed films were digitally photographed and subjected to densitometric analysis using GeneSnap software (Synoptics Ltd, Cambridge, UK).

**Figure 1.**
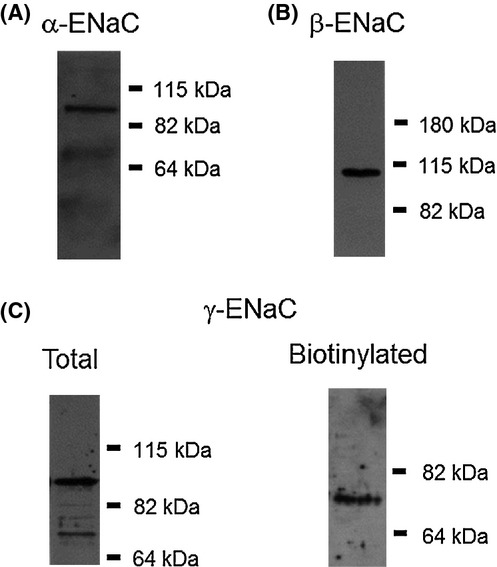
Specificity of ENaC antibodies. (A) In total and biotinylated samples the *α*‐ENaC antibody specifically recognized a band of approximately 95 kDa which is the molecular weight predicted for the full unprocessed form of the subunit. (B) In total and biotinylated samples, the *β*‐ENaC antibody specifically recognized a band of approximately 110 kDa which is the predicted molecular weight of the subunit. (C) For total samples the *γ*‐ENaC antibody detected two bands one of approximately 93 kDa, probably corresponding to the full length form, and other of approximately 75 kDa which could correspond to either the cleaved or the full unglycosylated form of the subunit. In biotinylated samples, the antibody specifically detected a band of 75 kDa.

The ENaC antibodies used in our study have been reported by others as specific for the different ENaC subunits (Butterworth et al. 2005a,b; Zhou et al. 2006; Portela‐Gomes et al. 2008; Chen et al. 2009; Li et al. 2009; Rubenstein et al. 2011). Moreover, our Western blots demonstrated the predicted molecular weight for all the different ENaC subunits tested here (Fig. 1).

### Biotinylation

M1‐CCD cells were grown on inserts of 4.2 cm^2^ area (Millipore, Hanging Cell Culture Inserts) until a transepithelial resistance >2 kΩ cm^2^ was reached. After E2 treatment, cell monolayers were washed twice with ice‐cold PBS and placed on ice. Sulfo‐NHS‐Biotin (Fisher Scientific Ireland, Dublin, Ireland) solution was prepared in PBS at a concentration of 1 mg/mL and applied on the apical side. Cells were incubated with this solution twice for 15 min on ice and then washed with PBS, followed by 15 min incubation with 100 mmol/L glycine. Cells were then washed with PBS and lysed. The amount of protein in each sample was quantified and between 300 and 500 *μ*g protein was incubated with streptavidin‐agarose beads (Fisher Scientific Ireland) overnight at 4°C. The next day samples were centrifuged for 2 min (2500*g* at 4°C), the supernatant was discarded and pellets were washed in lysis buffer and centrifuged again for 2 min (2500*g* at 4°C). The supernatant was discarded and pellets were resuspended in 40 *μ*L 2× Laemmli buffer. Forty microliters of biotinylated samples and 20–40 *μ*g of total lysate were run on SDS‐PAGE and then probed according to the Western blotting protocol described above.

### Immunofluorescence and confocal microscopy

M1‐CCD cells were grown on translucent inserts of 0.3 cm^2^ area (Millipore, Hanging Cell Culture Inserts) until a transepithelial resistance >0.6 kΩ cm^2^ was reached. After the end of each experiment cells were washed in ice‐cold PBS and apically labeled with Alexa Fluor 633 conjugated to wheat germ agglutinin (Alexa Fluor 633‐WGA, Invitrogen Biosciences Ltd, Dunlaoire, Ireland) (dilution 1:200 in PBS) for 10 min at 4°C. Labeling of the apical membrane with Alexa Fluor 633‐WGA was performed in advance for cell membrane disruption in order to stain the apical membrane only. Then, cells were fixed in 4% paraformaldehyde in PBS and permeabilized in 0.2% Triton X‐100 in PBS. The nonspecific binding of antibodies and fluorescent conjugates were blocked by incubation in 2% BSA in PBS for 1 h. Primary antibodies used were rabbit anti‐*α*‐ENaC (dilution 1:50) (Millipore, Cat. # AB3530P), rabbit anti‐*β*‐ENaC (dilution 1:50) (Abcam, Cat. # ab2906), and rabbit anti‐*γ*‐ENaC (dilution 1:50) (Abcam, Cat. # ab3468). Bound primary antibodies were detected using a goat anti‐rabbit Alexa Fluor 488 conjugate (Invitrogen Biosciences Ltd). Cells were mounted in Vectashield (Vector Laboratories, Peterborough, UK) containing 4′,6‐diamidino‐2‐phenylindole (DAPI), and examined using a Zeiss LSM 710 Meta confocal microscope. Laser excitation wavelengths for DAPI, Alexa Fluor 488, and Alexa Fluor 633 were 361, 488, and 633 nm, respectively. Images were captured at 63× magnification.

### Transepithelial ion transport studies

M1‐CCD cells were grown on inserts exposing an area of 0.6 cm^2^ (Millipore, Hanging Cell Culture Inserts) until reach a transepithelial resistance >0.6 kΩ cm^2^ and then mounted in Ussing chambers (Physiologic Instruments, San Diego, CA). The transepithelial potential difference (Vt) was clamped to 0 mV using an EVC‐4000 Voltage/Current clamp apparatus (World Precision Instruments). The transepithelial short‐circuit current (Isc) required to clamp Vt at 0 mV was recorded using Ag‐AgCl electrodes in 3 mol/L KCl agar bridges. Apical and basolateral baths were filled with Krebs's solution (in mmol/L: 115 NaCl, 25 NaHCO_3_, 0.4 KH_2_PO_4_, 2.4 K_2_HPO_4_, 1.2 MgCl_2_, 1.2 CaCl_2_, 10 glucose), pH 7.4 maintained at 37°C with a 95% O_2_/5% CO_2_ mixture. All mounted monolayers were allowed to equilibrate for 30–45 min prior to the experiments being performed. The Isc was defined as positive for cation flow from the apical to basolateral chamber. The current flowing through ENaC (I_Na_) was recorded as the amiloride‐inhibitable Isc. To investigate the Na^+^/K^+^‐ATPase electrogenic pump activity, the apical membrane was permeabilized with amphotericin B (50 *μ*mol/L) and NaCl was replaced by an equimolar concentration of *N*‐methyl‐d‐glucamine‐Cl^−^ (NMDG‐Cl^−^). Under these conditions, the ouabain‐sensitive Isc provides a measure of the current generated by the Na^+^/K^+^‐ATPase pump.

### Data analysis

The densitometry for phospho‐PKC*δ* and *α*‐, *β*‐, and *γ*‐ENaC subunit bands were normalized to the loading control *β*‐actin. All data are reported as mean ± SEM. Statistical analysis of the data was performed using a paired Student's *t*‐test for analysis between two groups. *P*‐values ≤0.05 were considered significant. One‐way ANOVA was used for multiple analyses of more than two groups with the Tukey post hoc test.

## Results

### Estrogen increases amiloride‐sensitive Isc in M1‐CCD cells

**Figure 2.**
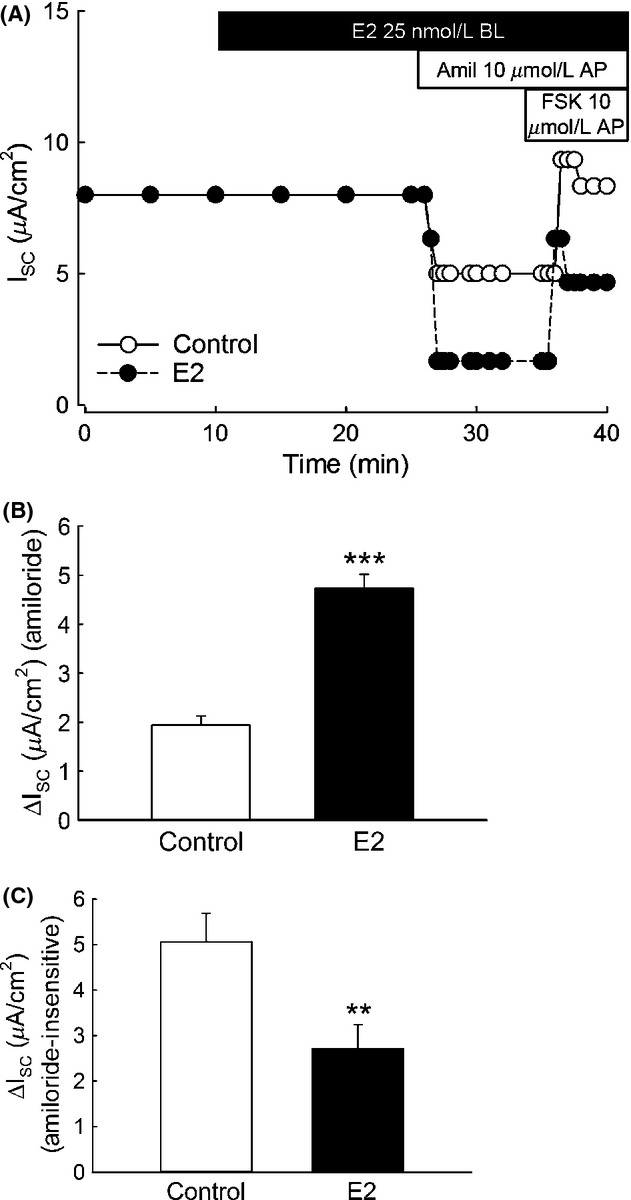
E2 increases the amiloride‐sensitive Isc in M1‐CCD cells. Ussing chamber experiments revealed that treatment with E2 (25 nmol/L) acutely (15 min) increased the amiloride‐sensitive Isc in polarized epithelia of M1‐CCD cells. (A) Representative Ussing chamber experiment showing the effect of E2 on the amiloride‐sensitive Isc (open circles control conditions, close circles E2 treated). (B) Graph showing mean values for the amiloride‐sensitive Isc for control and E2‐treated conditions (control, 1.9 ± 0.2 *μ*A/cm^2^; E2, 4.7 ± 0.3 *μ*A/cm^2^; *n* = 43, ***P < 0.001). (C) Graph showing values for the amiloride‐insensitive Isc (control, 5.1 ± 0.6 *μ*A/cm^2^; E2, 2.7 ± 0.5 *μ*A/cm^2^; *n* = 27, **P < 0.01)

**Figure 3.**
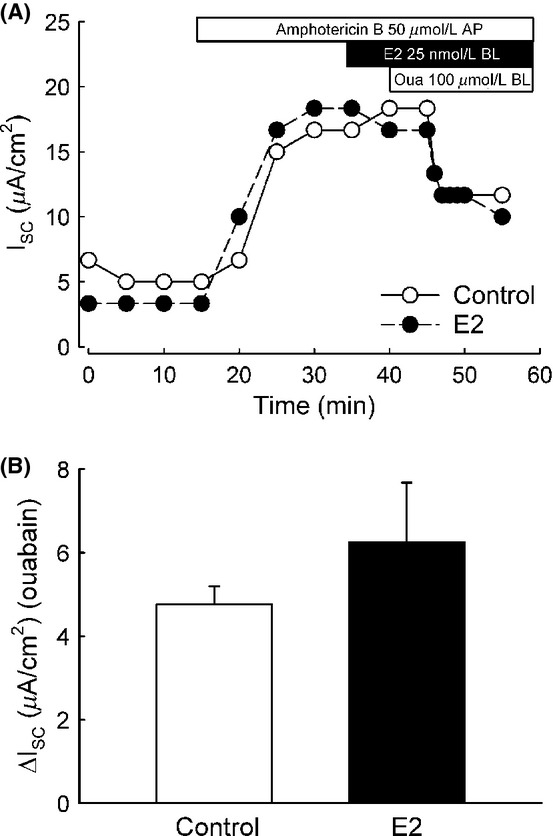
E2 does not increase the Na^+^/K^+^‐ATPase activity in M1‐CCD cells. Ussing chamber experiments in amphotericin B perforated epithelia using ouabain (100 *μ*mol/L) demonstrated that E2 does not affect the Na^+^/K^+^‐ATPase pump current. (A) Representative Ussing chamber experiment showing the effect of E2 in the ouabain‐sensitive Isc (open circles control conditions, close circles E2 treated). (B) Graph showing mean values for the ouabain‐sensitive Isc for control and E2‐treated conditions (control, 4.8 ± 0.4 *μ*A/cm^2^; E2, 6.3 ± 1.4 *μ*A/cm^2^; *n* = 4–7, *P* = 0.241).

We tested the acute effect of E2 on the amiloride‐sensitive Isc in polarized M1‐CCD cells. Basolateral addition of E2 (25 nmol/L) induced an increase in the amiloride (10 *μ*mol/L) ‐sensitive Isc within 15 min of treatment (Fig. 2A and B) and a decrease in the amiloride‐insensitive Isc (Fig. 2A and C). The activity of the Na^+^/K^+^‐ATPase pump, localized at the basolateral membrane in M1‐CCD cells, is critical to create the driving force necessary for the transepithelial movement of Na^+^ ions. Therefore, an increase in the Na^+^/K^+^‐ATPase activity could lead to an increase in the amiloride‐sensitive transepithelial Na^+^ currents. We investigated a possible effect of E2 on the activity of the Na^+^/K^+^‐ATPase as an explanation for the increase in the amiloride‐sensitive Isc observed in M1‐CCD cells. We found that E2, added basolaterally, failed to stimulate the activity of the Na^+^/K^+^‐ATPase, as determined from the ouabain‐sensitive current after permeabilization of the apical membrane with amphotericin B (50 *μ*mol/L) (Fig. 3A and B). This observation rules out the possibility of a direct acute regulation of the Na^+^/K^+^‐ATPase activity by E2 in M1‐CCD cells. Thus, it can be concluded that the stimulatory effect of E2 on the amiloride‐sensitive Isc is due to a direct regulation of ENaC and not secondary to changes in Na^+^/K^+^‐ATPase activity.

### Estrogen increases amiloride‐sensitive Isc via activation of PKC*δ*

**Figure 4.**
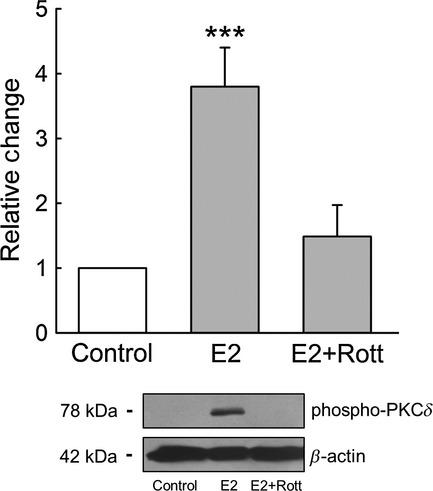
The stimulatory effect of E2 on PKC*δ* activity was inhibited by rottlerin. M1‐CCD monolayers were treated with rottlerin (5 *μ*mol/L) for 30 min prior to treatment with E2 (25 nmol/L) for 15 min. Cells were then harvested for protein extraction and subsequent Western blot analysis for phospho‐PKC*δ*. *Top*, densitometric analysis for the expression of phospho‐PKC*δ*;* bottom*, representative image of phospho‐PKC*δ* and *β*‐actin expression (*n* = 4, ***P < 0.001).

Previous work from our laboratory demonstrated that E2 treatment produces a rapid antisecretory response in rat colonic epithelia. This nongenomic effect was shown to be dependent on the activation of PKC*δ* (O'Mahony et al. 2007). On the basis of this observation, we investigated if PKC*δ* activation could be playing a role in the E2 proabsorptive response in CCD cells. E2 activation of PKC*δ* was tested using the inhibitor rottlerin. The reported IC_50_ for the inhibition of PKC*δ* by rottlerin is 3–6 *μ*mol/L (Gschwendt et al. 1994), and a concentration of 5 *μ*mol/L was used in our experiments. Caution must be exercised when interpreting results with PKC inhibitors, although rottlerin has been used as a PKC*δ* inhibitor in hundreds of studies, its specificity has been called into question in cell‐based assays (Soltoff 2007). That rottlerin inhibited PKC*δ* activation in our cell system was confirmed by the rottlerin inhibition of the E2‐induced PKC*δ* phosphorylation (Fig. 4).

**Figure 5.**
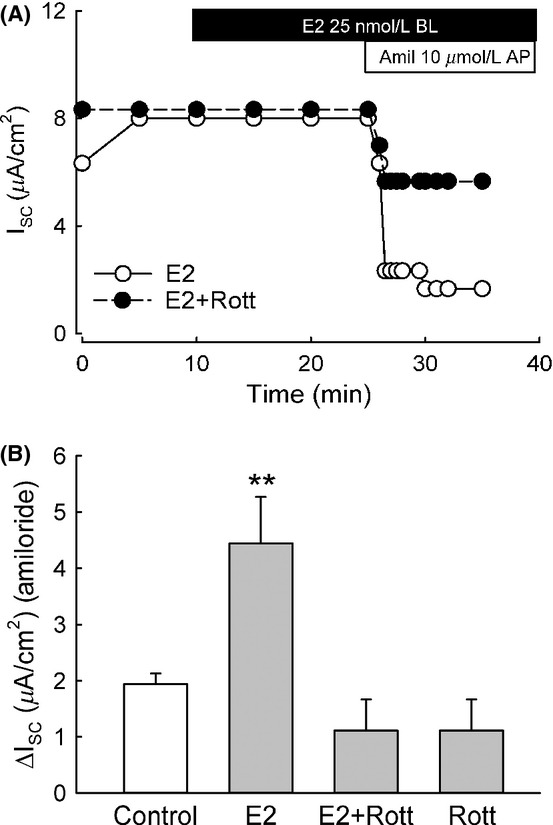
E2 increases the amiloride‐sensitive Isc in M1‐CCD cells via activation of PKC*δ*. M1‐CCD cells were pretreated for 30 min with the PKC*δ* inhibitor rottlerin (5 *μ*mol/L). (A) Representative Ussing chamber experiment showing the effect of E2 on the amiloride‐sensitive Isc (open circles, E2 treated; close circles, E2 treated + rottlerin). (B) Graph showing mean values for the amiloride‐sensitive Isc in the different conditions (control, 1.9 ± 0.2 *μ*A/cm^2^; rottlerin alone, 1.1 ± 0.5 *μ*A/cm^2^; E2, 4.4 ± 0.8 *μ*A/cm^2^; E2 + rottlerin, 1.1 ± 0.6 *μ*A/cm^2^; *n* = 6, **P < 0.01).

**Figure 6.**
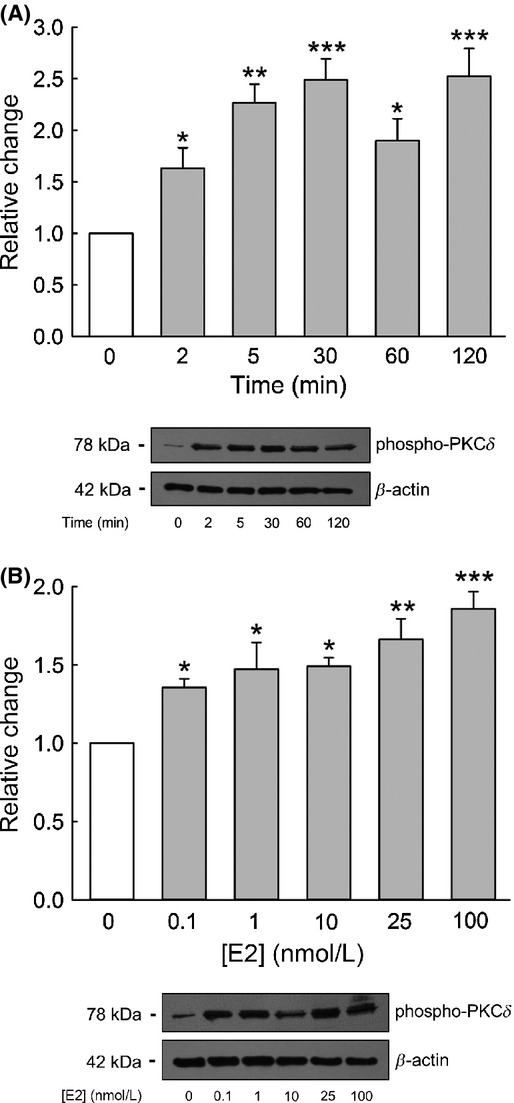
Time and dose dependence of PKC*δ* activation by E2. Western blot showing PKC*δ* autophosphorylation at residue Ser643 following E2 treatment. (A) Time dependence (*n* = 4, *P < 0.05, **P < 0.01, and ***P < 0.001 compared with the control). (B) Dose dependence (*n* = 4, *P < 0.05, **P < 0.01, and ***P < 0.001 compared with the control).

We found that inhibition of PKC*δ* with rottlerin abolished the increase in amiloride‐sensitive Isc induced by E2 (Fig. 5A and B). These results indicate the involvement of PKC*δ* in transducing the stimulatory effect of E2 on ENaC in CCD cells. We therefore determined the PKC*δ* activation sensitivity to E2 from changes in the PKC*δ* phosphorylation state at residue Ser643. E2 rapidly induced the activation of PKC*δ* within 2 min which was sustained over 2 h (Fig. 6A). The response was significant at low concentrations well within the physiological range of plasma levels of estrogen (0.1–0.8 nmol/L) (Fig. 6B).

### Signaling pathways involved in E2‐induced PKC*δ* activation

**Figure 7.**
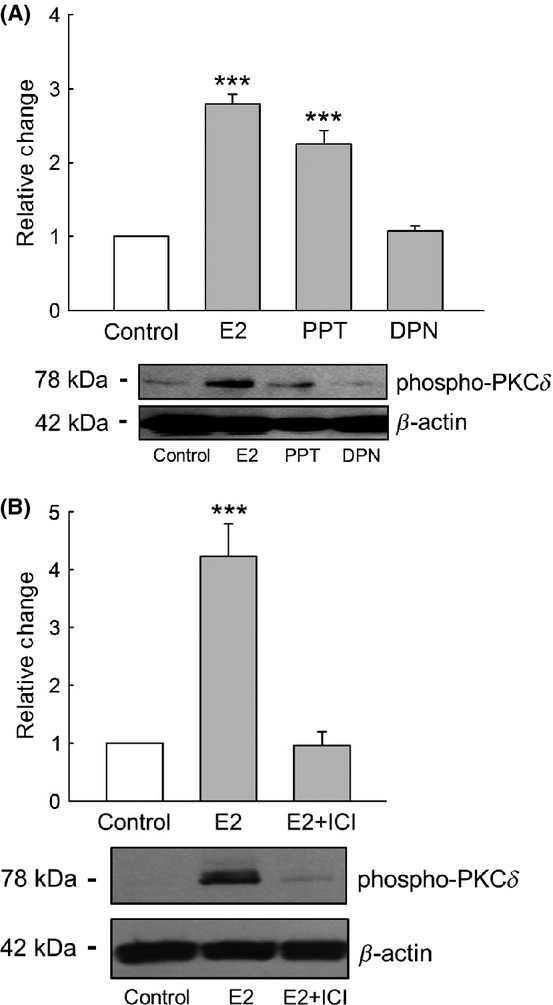
E2 activates PKC*δ* through a mechanism involving ER*α*. (A) PKC*δ* activity was stimulated by the ER*α* selective agonist PPT (1 nmol/L) but not by the ER*β* agonist DPN (5 nmol/L) (*n* = 4, ***P < 0.001 compared with the control). (B) E2 failed to stimulate the activity of PKC*δ* when M1‐CCD cells were pretreated for 30 min with the general ER antagonist ICI‐182780 (100 nmol/L) (*n* = 4, ***P < 0.001 compared with the control).

In breast cancer cells PKC*δ* activity can be stimulated by E2 through activation of matrix metalloproteinases (MMPs) followed by the release of membrane‐bound heparin‐binding epidermal growth factor (HB‐EGF) and trans‐activation of the epidermal grow factor receptor (EGFR) (Filardo et al. 2000). Here, we investigated the possibility that PKC*δ* can be activated in a similar fashion in CCD cells. First, we performed experiments where M1‐CCD cells were treated for 30 min with the estrogen receptor alpha (ER*α*) agonist PPT (1 nmol/L) or the estrogen receptor beta (ER*β*) agonist DPN (5 nmol/L). We found that PPT but not DPN activated PKC*δ* in a similar fashion to E2 (Fig. 7A), indicating that ER*α* is the receptor transducing PKC*δ* activation by E2 in CCD cells. Moreover, pretreatment of the CCD cell cultures with the general ER antagonist ICI‐182780 (100 nmol/L) inhibited the stimulatory effect of E2 on the activity of PKC*δ* (Fig. 7B), reaffirming the involvement of at least one classical ER isoform in the process.

**Figure 8.**
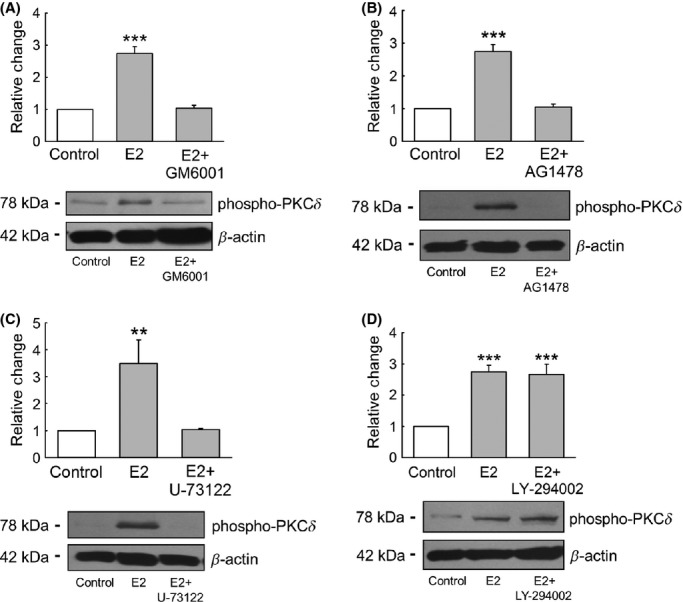
The MMP‐EGFR signaling pathway is involved in the activation of PKC*δ* induced by E2. (A) E2 failed to stimulate the activation of PKC*δ* when M1‐CCD cells were pretreated for 30 min with the MMP inhibitor GM6001 (1 *μ*mol/L) (*n* = 4, ***P < 0.001 compared with the control). Similar results were observed for experiments where M1‐CCD cells were pretreated for 30 min with (B) the EGFR inhibitor AG1478 (10 *μ*mol/L) (*n* = 4, ***P < 0.001 compared to the control conditions) and (C) the PLC inhibitor U‐73122 (10 *μ*mol/L) (*n* = 4, **P < 0.01 compared with the control). (D) Pretreatment for 30 min with the PI3K inhibitor LY‐294002 (25 *μ*mol/L) did not abolish the stimulatory effect of E2 on PKC*δ* activity (*n* = 4, ***P < 0.001 compared with the control).

We next explored the possible involvement of MMP‐EGFR and downstream signaling pathways in the E2 activation of PKC*δ*. Pretreatment of M1‐CCD cells for 30 min with the broad spectrum inhibitor for MMPs GM6001 (1 *μ*mol/L), produced an antagonistic effect on E2 activation of PKC*δ* (Fig. 8A). Similar inhibition of the E2 stimulatory effect on PKC*δ* was obtained when M1‐CCD cells were pretreated either with the EGFR inhibitor AG1478 (10 *μ*mol/L) (Fig. 8B) or with the PLC inhibitor U‐73122 (10 *μ*mol/L) (Fig. 8C). However, we found that pretreatment with the PI3K inhibitor LY‐294002 (25 *μ*mol/L) did not abolish the stimulation of PKC*δ* activity observed after acute treatment with E2 (Fig. 8D). Taken together, these results suggest that the activation of PKC*δ* induced by E2 in CCD cells is mediated through the MMP‐EGFR‐PLC signaling pathway.

### ER*α* is involved in the E2 stimulation of ENaC

**Figure 9.**
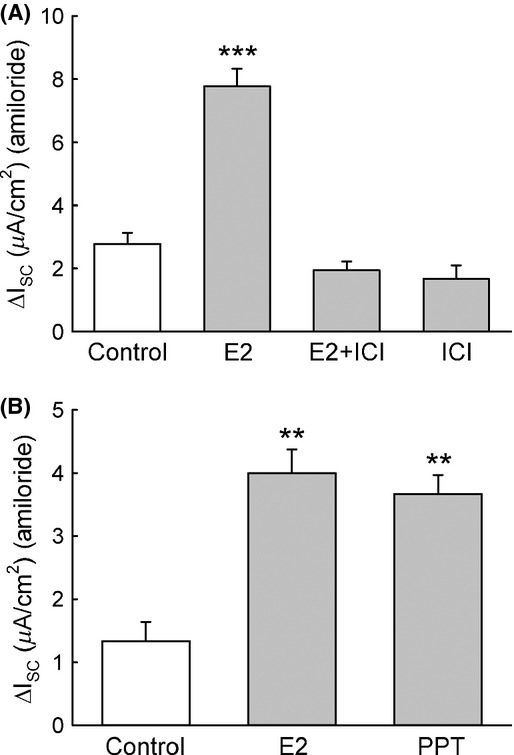
ER*α* is involved in the stimulation of the amiloride‐sensitive Isc induced by E2. (A) When M1‐CCD cells where pretreated for 30 min with the ER antagonist ICI‐182780 (100 nmol/L) there was no stimulation of the amiloride‐sensitive Isc by E2 (control, 2.8 ± 0.4 *μ*A/cm^2^; E2, 7.8 ± 0.6 *μ*A/cm^2^; E2 + ICI, 1.9 ± 0.3 *μ*A/cm^2^; ICI alone, 1.7 ± 0.4 *μ*A/cm^2^; *n* = 6, ***P < 0.001). Notice that ICI‐182780 alone, which has GPER agonist activity, failed to stimulate the amiloride‐sensitive Isc, indicating that GPER is unlikely to be involved in this process. (B) Treatment with the ER*α* agonist PPT (1 nmol/L for 15 min) increased the amiloride‐sensitive Isc in a similar fashion to E2 in polarized M1‐CCD cells epithelia (control, 1.3 ± 0.3 *μ*A/cm^2^; E2, 4.0 ± 0.4 *μ*A/cm^2^; PPT, 3.7 ± 0.3 *μ*A/cm^2^; *n* = 5, **P < 0.01).

The results presented above suggest that the activation of PKC*δ* is a necessary step for the stimulation of the amiloride‐sensitive Isc after acute treatment with E2; and also indicate that the activation of ER*α* is required for the stimulatory effect exerted by E2 on PKC*δ* activity. Thus, we hypothesized that ER activation could be required for the PKC*δ*‐dependent stimulatory effect of E2 on ENaC. We found that ER inhibition with the antiestrogen ICI‐182780 (100 nmol/L) for 30 min abolished the E2 effect on the amiloride‐sensitive Isc (Fig. 9A). Furthermore, treatment with the specific ER*α* agonist PPT (1 nmol/L) for 15 min had a similar effect to that observed following E2 treatment (Fig. 9B). These results suggest that the activation of ER*α* is an essential step in the stimulation of ENaC activity by E2.

### E2 activation of ENaC is transduced via MMP, EGFR, and PLC signaling

**Figure 10.**
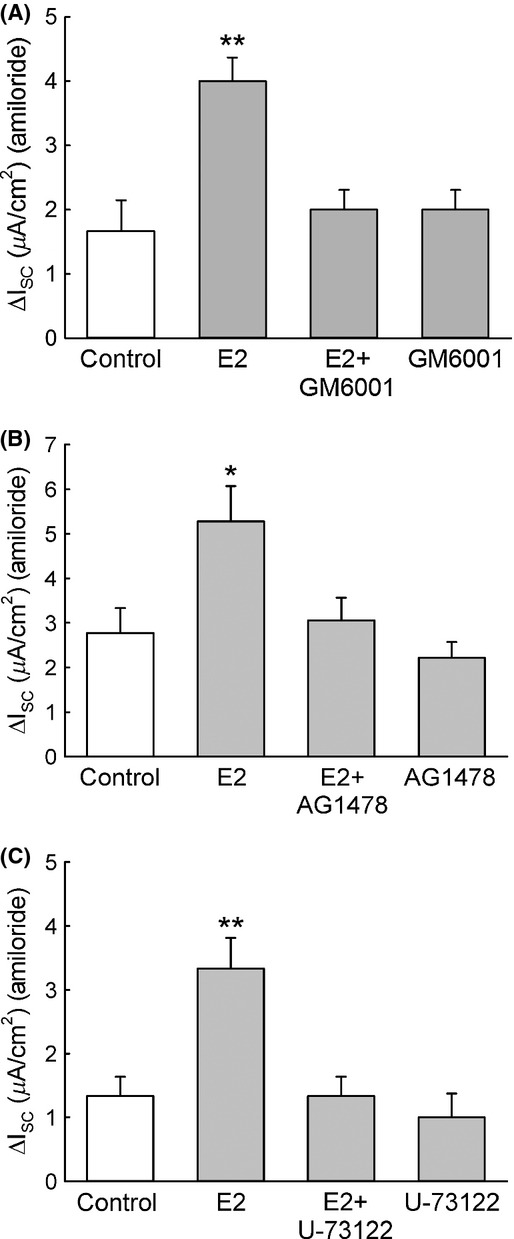
The increase in the amiloride‐sensitive Isc induced by E2 depends on MMP activation, EGFR transactivation, and PLC activation. (A) Ussing chamber experiments revealed that when M1‐CCD cells were pretreated for 30 min with the MMP inhibitor GM6001 (1 *μ*mol/L) there was no stimulation of the amiloride‐sensitive Isc induced by E2 (control, 1.7 ± 0.5 *μ*A/cm^2^; GM6001 alone, 2.0 ± 0.3 *μ*A/cm^2^; E2, 4.0 ± 0.4 *μ*A/cm^2^; E2 + GM6001, 1.9 ± 0.3 *μ*A/cm^2^; *n* = 5, **P < 0.01). Similar results were observed for Ussing chamber experiments were M1‐CCD cells were pretreated for 30 min with (B) EGFR inhibitor AG1478 (10 *μ*mol/L) (control, 2.8 ± 0.6 *μ*A/cm^2^; AG1478 alone, 2.2 ± 0.4 *μ*A/cm^2^; E2, 5.3 ± 0.8 *μ*A/cm^2^; E2 + AG1478, 3.1 ± 0.5 *μ*A/cm^2^; *n* = 6, *P < 0.05) or (C) PLC inhibitor U‐73122 (10 *μ*mol/L) (control, 1.3 ± 0.3 *μ*A/cm^2^; U‐73122 alone, 1.0 ± 0.4 *μ*A/cm^2^; E2, 3.3 ± 0.5 *μ*A/cm^2^; E2 + U‐73122, 1.3 ± 0.4 *μ*A/cm^2^; *n* = 5, **P < 0.01).

As the increase in the amiloride‐sensitive Isc observed after the acute treatment with E2 depends on PKC*δ* activation, which is coupled to a signaling cascade including the MMPs, EGFR, and PLC, we tested whether these three signaling components also participate in the estrogen stimulation of the amiloride‐sensitive Na^+^ absorption in CCD cells. E2 treatment failed to activate ENaC when M1‐CCD cells were pretreated for 30 min with the MMP inhibitor GM6001 (1 *μ*mol/L) (Fig. 10A). Similar inhibitory effects on the ENaC current response to E2 were observed after treatment with the EGFR inhibitor AG1478 (10 *μ*mol/L) (Fig. 10B) or the PLC inhibitor U‐73122 (Fig. 10C). These results further confirm the involvement of the MMP signaling cascade, EGFR and PLC, not only in the activation of PKC*δ* but also in the ENaC activation induced by E2.

### Nuclear‐excluded estrogen bound ER activates ENaC in M1‐CCD cells

**Figure 11.**
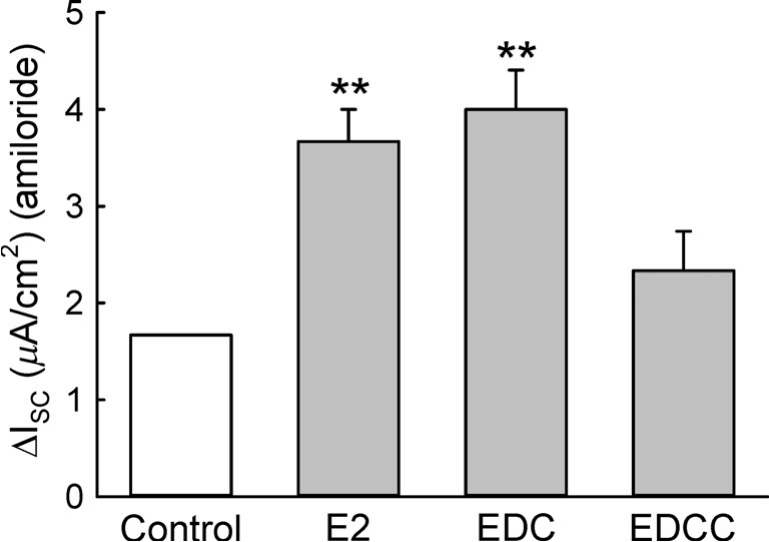
EDC increases the amiloride‐sensitive Isc. Ussing chamber experiments where M1‐CCD cells were treated with EDC at a final concentration equivalent to 25 nmol/L of free E2. EDC, but not the unconjugated dendrimer (EDCC), increased the amiloride‐sensitive Isc comparable to E2 (control, 1.7 ± 0.1 *μ*A/cm^2^; E2, 3.7 ± 0.3 *μ*A/cm^2^; EDC, 4.0 ± 0.4 *μ*A/cm^2^; EDCC, 2.3 ± 0.4 *μ*A/cm^2^; *n* = 5, **P < 0.01).

Given the rapid response to E2, we reasoned that the hormone effects on PKC*δ* and ENaC were primarily nongenomic in onset (the sustained responses may involve transcriptional events). To test this hypothesis, we conducted studies using an E2 dendrimer conjugate which when bound to the estrogen receptor prevents its translocation to the nucleus. In the past, E2 conjugated to membrane impermeable carriers such as BSA have been used to support the existence of membrane‐associated ERs and nongenomic responses. Criticism surrounding the stability of these conjugates following internalization led to the development of more stable conjugates such as the estrogen conjugate dendrimer (EDC) (Harrington et al. 2006). This compound is composed of a dendrimer molecule (PAMAM) that is coupled to ~20 molecules of estrogen, allowing the EDC to interact at the plasma membrane and enter the cytoplasm but not penetrate the nuclear envelope, thus discriminating the genomic from the nongenomic effects of E2 (Harrington et al. 2006). The effects of EDC on the amiloride‐sensitive Isc were compared with free estrogen. Ussing chamber experiments where M1‐CCD cells were treated for 15 min with EDC at a final concentration equivalent to 25 nmol/L of free E2 demonstrated that EDC stimulates ENaC activity in a similar manner to unbound E2 (Fig. 11). These results point to a rapid “nongenomic” response to estrogen transduced at the membrane or in the cytosol by the estrogen receptor ER*α* without requiring the receptor–ligand complex entering the nucleus. This observation does not exclude other later transcriptional events occurring via the E2 activated EGFR pathway to cause sustained activation of ENaC.

### Estrogen rapidly increases the apical membrane abundance of the *γ*‐ENaC subunit in M1‐CCD cells

**Figure 12.**
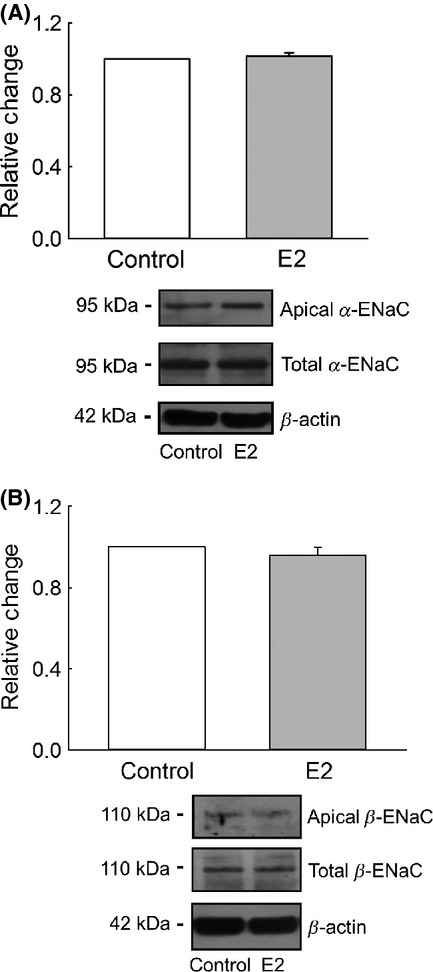
Acute treatment with E2 did not change the apical membrane abundance of *α*‐ or *β*‐ENaC. M1‐CCD cells grown on permeable supports were basolaterally treated with E2 (25 nmol/L) for 15 min. The apical abundance of *α*‐ and *β*‐ENaC was assessed by biotinylation of apical proteins and subsequent Western blotting using specific antibodies against *α*‐ or *β*‐ENaC as indicated. (A) *Top*, densitometric analysis for the apical abundance of *α*‐ENaC; *bottom*, a representative image for the biotinylated and total *α*‐ENaC expression as well as the expression of *β*‐actin (*n* = 4, *P* = 0.405). (B) *Top*, densitometric analysis for the apical expression of the *β*‐ENaC; *bottom*, a representative image for the biotinylated and total *β*‐ENaC expression, the expression of *β*‐actin is also shown (*n* = 6, *P* = 0.350).

**Figure 13.**
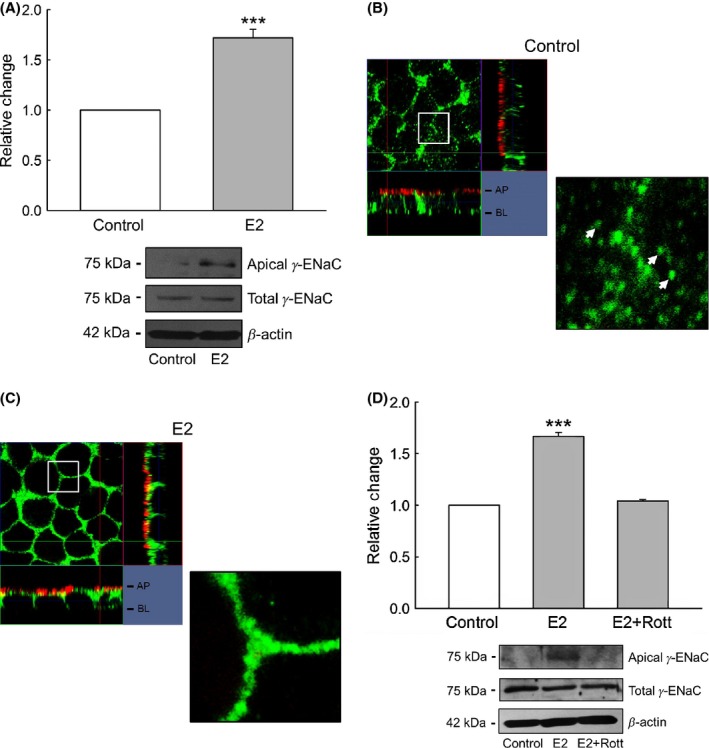
E2 rapidly increases the apical membrane surface abundance of *γ*‐ENaC. (A) Biotinylation experiments revealed that after 30 min treatment with E2, there was a significant increase in the apical surface abundance of *γ*‐ENaC (*n* = 4, ***P < 0.001). In this set of experiments the antibody specifically detected a band of approximately 75 kDa, corresponding to the cleaved form of *γ*‐ENaC. Immunostaining and confocal microscopy experiments using a specific antibody recognizing *γ*‐ENaC (green) and the plasma membrane (red) confirmed these biotinylation results. (B) In control conditions, *γ*‐ENaC was found in clusters present in the cytosol, the close proximity to the plasma membrane (white arrows) and in the plasma membrane. (C) After acute treatment with E2, the *γ*‐ENaC fluorescence signal was predominately found in the plasma membrane. Confocal Z‐stack images are representative of three independent experiments. (D) Pretreatment for 30 min with the PKC*δ* inhibitor rottlerin (5 *μ*mol/L) abolished the increase in the apical surface abundance of *γ*‐ENaC after treatment with E2 (*n* = 4, ***P < 0.001).

A plausible explanation for the observed rapid increase in the amiloride‐sensitive Isc in M1‐CCD cells after treatment with E2 could result from changes in the abundance of the different ENaC subunits at the apical plasma membrane, similar to that described for the nongenomic effects of aldosterone on ENaC subunits membrane abundance in M1‐CCD cells (McEneaney et al. 2010). To test our hypothesis, we looked for changes in the total abundance and surface expression of *α*‐, *β*‐, and *γ*‐ENaC subunits using biotinylation, Western blotting, and immunofluorescence. This analysis revealed that after 30 min treatment with E2, the *α*‐ and *β*‐ENaC subunits did not change either their total expression or apical membrane abundance (Fig. 12). The *γ*‐ENaC total expression was also not significantly changed in response to E2 treatment; however, there was a significant increase in the *γ*‐ENaC apical surface abundance (Fig. 13A). These results were confirmed by immunolocalization experiments (Fig. 13B and C).

Finally, we investigated whether PKC*δ* activation was involved in the acute increase in the apical surface abundance of the *γ*‐ENaC subunit following E2 treatment. Inhibition of PKC*δ* using rottlerin (5 *μ*mol/L) for 30 min abolished the increase in the apical membrane abundance of *γ*‐ENaC normally observed with E2 (Fig. 13D).

These results suggest that the mechanism by which E2 stimulates the amiloride‐sensitive Isc in M1‐CCD cells is related to its stimulatory effect on the abundance of *γ*‐ENaC at the apical membrane and not to changes in ENaC subunit protein expression. This process is dependent on the activation of PKC*δ* which plays a role in the intracellular trafficking of *γ*‐ENaC to the apical plasma membrane following E2 treatment.

## Discussion

The normal functioning of the kidney is central to maintaining fluid and electrolyte homeostasis of the whole body. In the distal parts of the nephron, the activity of ENaC is the rate‐limiting step for Na^+^ reabsorption and accounts for the fine‐tuning of Na^+^ content of the whole body. This process is tightly regulated by steroidal and nonsteroidal hormones such as aldosterone, vasopressin, and insulin. The main aim of this study was to determine whether E2 had an effect on Na^+^ reabsorption in cortical collecting duct using M1‐CCD cells. We found a completely novel action of estrogen to rapidly increase the ENaC current in CCD cells through a nongenomic mechanism involving cytosolic or membrane‐associated ER*α*, MMP‐dependent EGFR transactivation followed by PLC activation, and PKC*δ* phosphorylation to induce an increase in the abundance of the *γ*‐ENaC subunit at the apical membrane.

Aside from the rapidity of the response, our results are qualitatively similar to the observations of chronic long‐term E2 treatment in alveolar and kidney cells (Sweezey et al. 1998; Gambling et al. 2004). However, one study found no estrogen effect in alveolar epithelial cells (Laube et al. 2011), where treatment with E2 alone for between 48 and 72 h did not induce a significant increase in amiloride‐sensitive currents; although it was also shown that E2 and progesterone together increase the amiloride‐sensitive currents and mRNA levels for the *α*‐ and *β*‐ENaC subunits (Laube et al. 2011). Our data indicate that the mechanism involved in the increase in amiloride‐sensitive currents induced by E2 in M1‐CCD cells does not require progesterone. It cannot be ruled that these differences are due to a different cellular culture system which could affect expression of the estrogen receptor, progesterone receptor, or other proteins participating in the signaling pathway.

The rapid E2 effect on ENaC activity suggests a mechanism involving a nongenomic action that may augment the transcriptional response in some tissues. The evidence for a nongenomic response was strengthened by the observation of an increase in the amiloride‐sensitive current after treatment with the estrogen dendrimer compound EDC, which has been demonstrated to be a useful tool to dissect genomic and nongenomic effects of E2 (Harrington et al. 2006). The implication is that the early ENaC response to estrogen is initiated by a signaling event at the cell membrane. Therefore, this process seems to be different in nature from those previously described in the rat lung epithelia (Sweezey et al. 1998) and rat kidney of female rats (Gambling et al. 2004); where in both cases the increase in the amiloride‐sensitive current in response to E2 is associated with increase in the mRNA levels of *α*‐ and *γ*‐ENaC subunits at least 8 h after treatment implying a genomic effect (Horisberger 2003).

**Figure 14.**
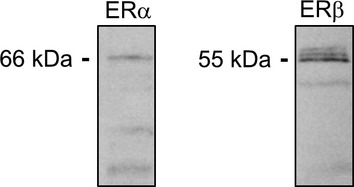
M1‐CCD cells monolayers express ER*α* and ER*β* at the protein level. M1‐CCD cell monolayers were harvested for protein extraction and subsequent Western blotting using specific antibodies against ER*α* and ER*β*, respectively.

It has been recently shown that E2 evokes intracellular Ca^2+^ transients and increases the H^+^‐ATPase activity in intercalated cells in mouse distal convoluted tubules, connecting tubules, and initial cortical collecting ducts via a G‐protein coupled estrogen receptor (GPER)‐dependent signaling pathway (Hofmeister et al. 2012). Our data, showing that ICI‐182780 (which is also a GPER agonist) did not stimulate the amiloride‐sensitive current, indicates that GPER does not participate in the nongenomic response to E2 in M1‐CCD cells. Moreover, our results strongly suggest that an ER*α* isoform transduces the rapid E2 effect on ENaC. Interestingly, a recent study has found that the three ER*α* isoforms are expressed in the kidney (Irsik et al. 2013). The same study showed that ER*α*36, the membrane‐associated isoform that mediates rapid effects of E2 in other tissues (Pedram et al. 2009, 2013), is highly expressed in the membrane fraction of the renal cortex (Irsik et al. 2013). In our study we found that M1‐CCD cells monolayers express ERa and ERb (Fig. 14).

**Figure 15.**
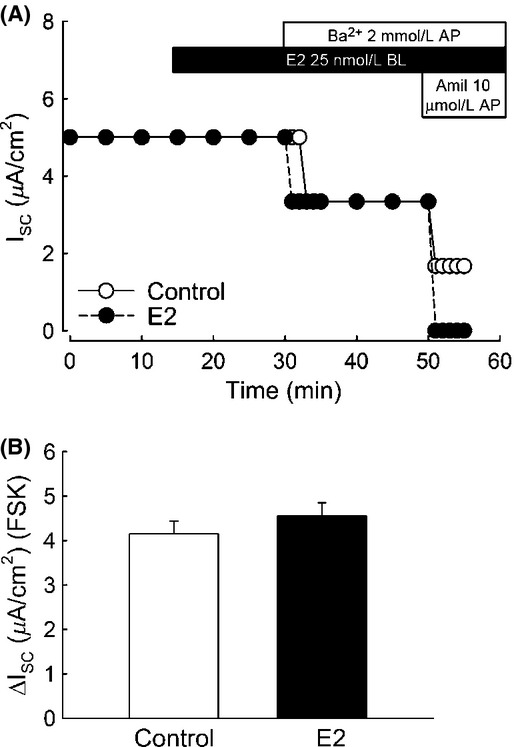
Effect of acute treatment with E2 on the Ba^2+^‐sensitive current in M1‐CCD polarized monolayers. (A) A representative tracing of changes in Isc in control conditions and after basolateral treatment of E2 (25 nmol/L) for 15 min, the responses after the apical addition of Ba^2+^ (2 mmol/L) and amiloride (10 *μ*mol/L) are also shown. (B) Values for the Ba^2+^‐sensitive Isc: Control, 2.1 ± 0.3 *μ*A/cm^2^; E2, 2.4 ± 0.7 *μ*A/cm^2^; *n* = 7, *P* = 0.765.

**Figure 16.**
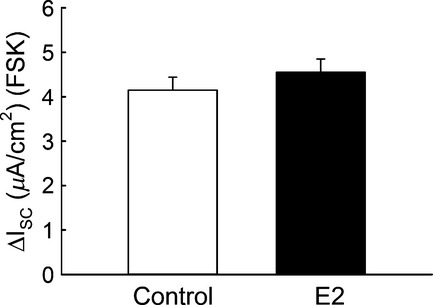
FSK‐stimulated Isc was not affected by the acute treatment with E2 in M1‐CCD polarized monolayers. Values for the FSK‐stimulated Isc: Control, 4.2 ± 0.3 *μ*A/cm^2^; E2, 4.6 ± 0.3 *μ*A/cm^2^; *n* = 41, *P* = 0.332.

E2 produced an increase in the amiloride‐sensitive component of the transepithelial current and a decrease in the amiloride‐insensitive component of the current in M1‐CCD cells. These effects appear to be specific for ENaC as both Ba^2+^‐sensitive and the FSK‐stimulated Isc were not affected by E2 (Figs. 15 and 16, respectively), thus ruling out K^+^ channels and cAMP‐activated Cl^−^ channels as estrogen targets in CCD cells. The expression of Ca^2+^‐activated Cl^−^ channels (CaCCs) has been found in different renal cell types (Rajagopal et al. 2012; Buchholz et al. 2014; Faria et al. 2014). The lack of transepithelial current response to carbachol observed in M1‐CCD cells under control and E2‐treated conditions (data not shown) suggest that CaCCs do not contribute to the effect of E2 on transepithelial ion transport in M1‐CCD cells.

**Figure 17.**
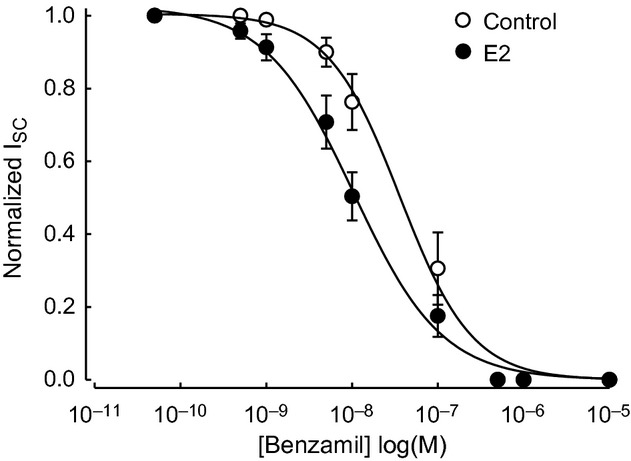
Benzamil dose–response curves. Increasing concentrations of benzamil were added to the apical side of polarized M1‐CCD cells under control (open circles) and E2‐treated (close circles) conditions. Changes in the amplitude of the Isc were recorded in Ussing chambers. The Isc was normalized to the basal Isc in the absence of benzamil. The calculated values for IC_50_ were 36.20 ± 6.98 nmol/L (control) and 10.45 ± 1.92 nmol/L (E2) (*n* = 9). The IC_50_ values were estimated by fitting the following equation *y* = 1/(1 + 10^[log IC50 − log X]^).

The effect of E2 to enhance the amiloride‐sensitive I_Na_ may be explained by the recent finding that ENaC channels composed of *α* and *β* subunits have a very high open probability but a reduced sensitivity to amiloride compared to those composed of either *α* and *γ* or the three ENaC subunits (Shi and Kleyman 2013). It is possible that estrogen converts ENaC channels with low sensitivity to amiloride (ENaC *αβ*) to a more amiloride‐sensitive phenotype (ENaC *αβγ*) given our findings of increased *γ* subunit abundance in the apical membrane after E2 treatment. A higher expression of ENaC *αβγ* channels could explain the apparent relative decrease in the amiloride‐insensitive component of the Isc. ENaC channels are more sensitive to benzamil than amiloride and we compared the dose–response of benzamil before and after estrogen treatment. A significant increase in the sensitivity of ENaC currents to benzamil was observed following E2 treatment (Fig. 17), suggesting that estrogen may enhance the abundance of ENaC *αβγ* channels in the apical membrane.

Previously published evidence suggests that PKC*α* activation leads to inhibition of ENaC activity (Stockand et al. 2000; Awayda et al. 2002; Booth and Stockand 2003). However, the results presented here and those published by others (Ali et al. 1998; Sun et al. 2012) suggest that PKC activation can also lead to stimulation of ENaC activity. This contradiction could be attributed to differences in the expression and/or activation profiles of the different PKC isozymes mediating these effects.

In previous studies we have demonstrated that E2 specifically activates PKC*δ* to produce the antisecretory response in distal colon (O'Mahony et al. 2007, 2009; O'Mahony and Harvey 2008). In these studies, we confirmed that rottlerin (up to a concentration of 10 *μ*m) did not inhibit PKC*α*, PKC*β*, or PKC*ε* activation by E2 and that inhibition of these PKC isozymes did not suppress the antisecretory response to E2. In this study we found that rottlerin inhibited the E2‐induced PKC*δ* autophosphorylation and activation in CCD cells (Fig. 4). Thus, in our study, a rottlerin inhibitory effect on secretion and absorption may be interpreted as specifically involving PKC*δ*.

The mechanism linking PKC*δ* activity to the increase in ENaC activity and apical surface expression of *γ*‐ENaC is unclear. However, it has been shown that PKC*δ* regulates intracellular trafficking of ENaC subunits in M1‐CCD cells through a mechanism dependent on the activity of PKD1 (McEneaney et al. 2008). PKD1 is a serine/threonine kinase that is activated through a nPKC‐dependent pathway (Gliki et al. 2002; Scaltriti and Baselga 2006) and has been implicated in intracellular trafficking through its function at the TGN to regulate the fission of vesicles specifically destined for the cell surface (Scaltriti and Baselga 2006). Thus, PKD1 could be the signaling component mediating the increase in ENaC activity and apical surface expression of *γ*‐ENaC subsequent to PKC*δ* activation by E2.

In this study, the *γ*‐ENaC antibody detected two bands, one of 93 kDa, corresponding to the full length form, and other of 75 kDa corresponding to either the cleaved or the fully unglycosylated form of the subunit. In the biotinylation experiments, the antibody specifically detected a band at 75 kDa, which probably corresponds to the cleaved form of the *γ*‐ENaC subunit (Fig. 1C). The *α*‐ENaC antibody detected only the full length form (95 kDa) of *α*‐ENaC (Fig. 1A). Hence, we were unable to test the possibility of a change in the proportion between the full and cleaved forms of either the *α*‐ or *γ*‐ENaC subunits at the apical membrane in response to E2. Nevertheless, the results from the immunostaining experiments suggest that E2 response is driven by stimulation of the trafficking of *γ*‐ENaC toward the apical membrane. It is most probable that E2 stimulates the trafficking of all ENaC subunits together to the membrane as channel formation is unlikely to occur through subunit complex aggregation in the membrane.

**Figure 18.**
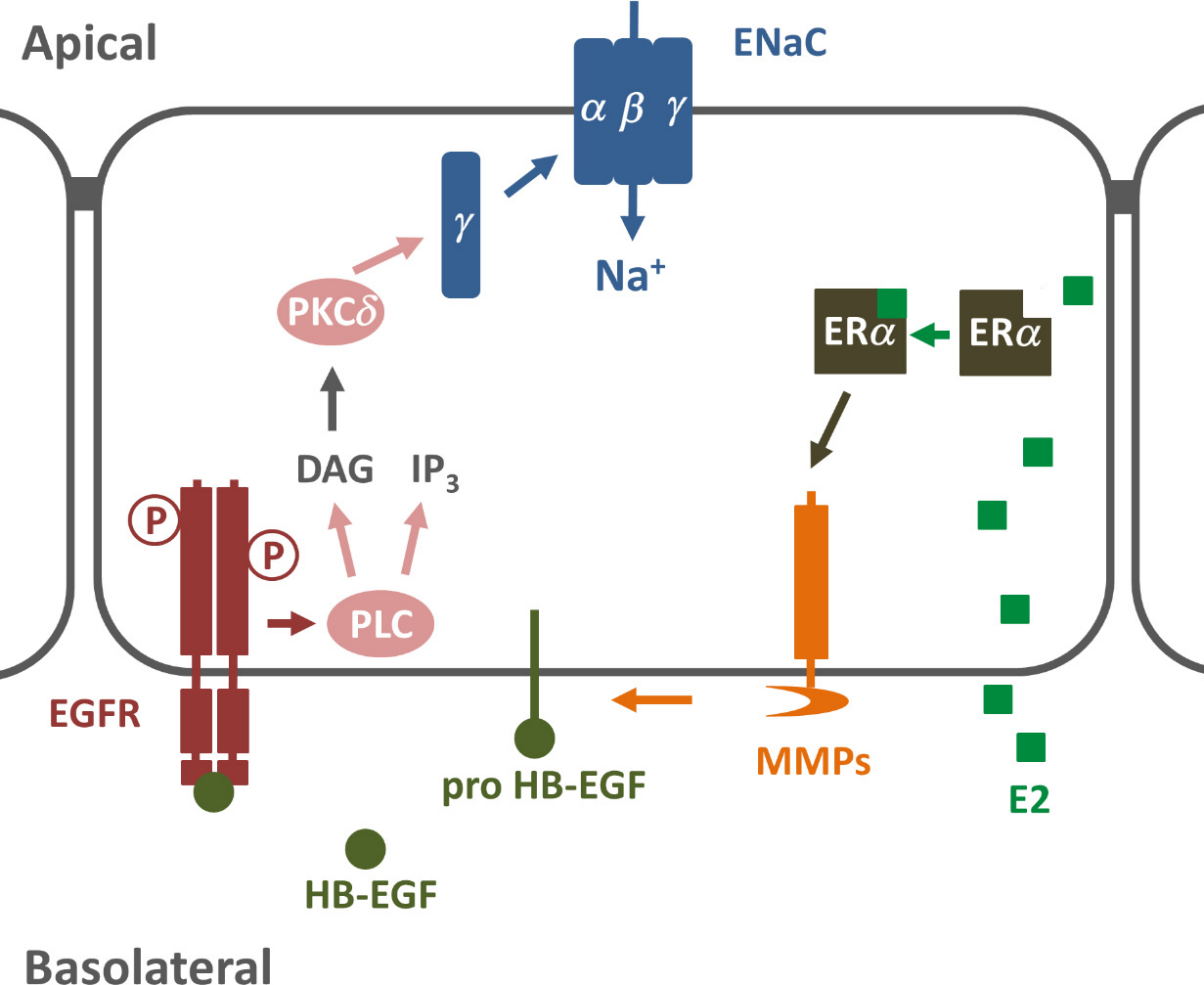
Schematic representation showing the proposed signaling pathways involved in activation of ENaC following acute treatment with E2 in cortical collecting duct M1‐CCD cells. Following the binding of E2 to ER*α*, the activation of MMPs stimulates the release of HB‐EGF. Next, HB‐EGF binds EGFR inducing the trans‐activation of the receptor and subsequent increase in the activity of PLC. The rise in DAG levels, produced by the activation of PLC, leads to activation of PKC*δ* which, in turn, increases the apical surface abundance of the *γ*‐ENaC subunit and the activity of ENaC in the apical membrane of CCD cells.

In conclusion, this study describes a novel function for estrogen as a salt‐retaining hormone in kidney cortical collecting duct cells and provides molecular mechanistic insights into its mode of action on ENaC in distal renal tubule cells (Fig. 18). The effects of estrogen on ENaC may be additive or separate from aldosterone regulation. The study provides evidence for the involvement of the “aldosterone‐sensitive CCD” to effect an increased whole body fluid volume observed in high estrogen states.

## Acknowledgments

The authors thank John Katzenellenbogen for supplying EDC.

## Conflict of Interest

None declared.
